# A Major Locus for Manganese Tolerance Maps on Chromosome A09 in a Doubled Haploid Population of *Brassica napus* L.

**DOI:** 10.3389/fpls.2017.01952

**Published:** 2017-12-12

**Authors:** Harsh Raman, Rosy Raman, Brett McVittie, Beverley Orchard, Yu Qiu, Regine Delourme

**Affiliations:** ^1^New South Wales Department of Primary Industries, Wagga Wagga Agricultural Institute, Wagga Wagga, NSW, Australia; ^2^INRA, Agrocampus Ouest, Université de Rennes 1, UMR1349 Institut de Génétique, Environnement et de Protection des Plantes, Le Rheu, France

**Keywords:** natural variation, linkage mapping, canola, physical mapping, candidate genes, acid soils

## Abstract

Soil acidity poses a major threat to productivity of several crops; mainly due to the prevalence of toxic levels of Al^3+^ and Mn^2+^. Crop productivity could be harnessed on acid soils via the development of plant varieties tolerant to phytotoxic levels of these cations. In this study, we investigated the extent of natural variation for Mn^2+^ tolerance among ten parental lines of the Australian and International canola mapping populations. Response to Mn^2+^ toxicity was measured on the bases of cotyledon chlorosis, shoot biomass, and leaf area in nutrient solution under control (9 μM of MnCl_2_⋅4H_2_O) and Mn treatment (125 μM of MnCl_2_⋅4H_2_O). Among parental lines, we selected Darmor-*bzh* and Yudal that showed significant and contrasting variation in Mn^2+^ tolerance to understand genetic control and identify the quantitative trait loci (QTL) underlying Mn^2+^ tolerance. We evaluated parental lines and their doubled haploid (DH) progenies (196 lines) derived from an F_1_ cross, Darmor-*bzh*/Yudal for Mn^2+^ tolerance. Mn^2+^-tolerant genotypes had significantly higher shoot biomass and leaf area compared to Mn^2+^-sensitive genotypes. A genetic linkage map based on 7,805 DArTseq markers corresponding to 2,094 unique loci was constructed and further utilized for QTL identification. A major locus, *BnMn*^2+^.*A09* was further mapped with a SNP marker, Bn-A09-p29012402 (LOD score of 34.6) accounting for most of the variation in Mn^2+^ tolerance on chromosome A09. This is the first report on the genomic localization of a Mn^2+^ tolerance locus in *B. napus*. Additionally, an ortholog of *A. thaliana* encoding for cation efflux facilitator transporter was located within 3,991 bp from significant SNP marker associated with *BnMn*^2+^.*A09*. A suite of genome sequence based markers (DArTseq and Illumina Infinium SNPs) flanking the *BnMn*^2+^.*A09* locus would provide an invaluable tool for various molecular breeding applications to improve canola production and profitability on Mn^2+^ toxic soils.

## Introduction

Canola (*Brassica napus* L., 2*n* = 4× = 38, genome = A_n_A_n_C_n_C_n_) is the second major oilseed crop grown worldwide, followed by soybean^1^. It is used as a source of healthy vegetable oil for human consumption, fodder and canola meal for animals, biodiesel for renewable energy and for other pharmaceutical applications (Friedt and Snowdon, 1999). Canola leaves and stems are also used for different culinary preparations such as ‘Saag’ especially in the Indian subcontinent. The increasing demand for canola especially for vegetable oil and biodiesel requires improved strategies to produce higher grain and biomass yield per unit area.

Soil acidity is a major limitation to crop production on 40% of the world’s arable land, caused mainly by the toxic levels of Al^3+^ and Mn^2+^ (von Uexküll and Mutert, 1995; Conyers et al., 1997). Of the Earth’s crust, manganese is the second most abundant transition metal after iron, and an essential micronutrient for plant growth (Marschner, 1995). At low pH (≤5), di- and trivalent cations (Mn^2+^ and Al^3+^) become solubilized into solution form and inhibit growth of sensitive plants (Pittman, 2005). Extreme environment conditions such as high temperatures in summer, and waterlogging at the end of winter, can also increase the levels of exchangeable Mn^2+^ in soil and increase the incidence of toxicity (Sparrow and Uren, 1987; Slattery and Ronnfeldt, 1992). Micronutrient deficiency of Fe and Zn can also enhance the uptake of Mn^2+^ in some plants (Cohen et al., 1998). Al^3+^ toxicity affects plant growth and yield potential mainly via inhibition of root-tips, whereas Mn^2+^ toxicity affects plant growth via disrupting photosynthesis due to extensive cotyledon and leaf (margin and interveinal) chlorosis, leaf crinkling/cupping, and leaf and root necrosis in Mn^2+^-sensitive plants (Heenan and Campbell, 1981; Nable et al., 1988; Moroni et al., 1991, 2003; Kassem et al., 2004). Soil acidity can be ameliorated by increasing the pH with the heavy surface application of lime or dolomite (Scott et al., 1997). However, this approach will not fully alleviate Mn^2+^ toxicity (Bromfield et al., 1983). In addition, it is very difficult to incorporate lime in the deeper layers of subsoils.

Generally, canola is not recommended for commercial cultivation on strong acidic soils (pH_CaCl2_ ≤ 4.5). However, crop productivity could be harnessed on acid soils via the development of plant varieties tolerant to phytotoxic levels of Mn^2+^. Considerable genetic variation, and its physiological and molecular bases for Al^3+^ tolerance have been revealed in various key agricultural crop plants such as wheat, barley, rice, and sorghum (Moroni et al., 1991; Raman et al., 2002, 2005, 2008; Wang et al., 2007; Ryan et al., 2009, 2010). Several studies have identified qualitative and quantitative loci associated with Al^3+^ tolerance (Wu et al., 2000; Magalhaes et al., 2007; Cai et al., 2008; Ryan et al., 2009; Krill et al., 2010). In addition, candidate genes involved in Al^3+^ tolerance have also been isolated and functionally characterized (Delhaize et al., 2004; Raman et al., 2005, 2008; Magalhaes et al., 2007; Ryan et al., 2010). However, information to such extent for Mn^2+^ tolerance is not yet available for many agricultural crops including canola.

Previous studies have shown that a natural variation for Mn^2+^ tolerance exists in thale cress (*Arabidopsis thaliana* L.), soybean (*Glycine max* L. Merr.), canola and common wheat (*Triticum aestivum* L.) (Foy, 1984; Hocking et al., 1999; Moroni et al., 2003; Kassem et al., 2004). Wratten and Scott (1979) showed the presence of natural variation for tolerance to Mn^2+^ toxicity in *B. napus* and *Brassica rapa.* In a subsequent study, Moroni et al. (2003) evaluated 39 germplasm accessions and reconfirmed Mn^2+^ tolerance in *B. napus*; ‘Mutu (Mutsu),’ ‘Wesreo,’ ‘Tower,’ and ‘91-215-3’ and in one accession of *B. rapa;* ‘Duro.’ Genetic analysis of the intercross population derived from Mutu-98-6 (Mn^2+^-tolerant)/RSO94-67 (Mn^2+^-sensitive) suggests that Mn^2+^ tolerance is genetically controlled by a single major locus (Moroni et al., 2003; McVittie et al., 2011). However, the locus conferring Mn^2+^ tolerance has not been mapped yet to the *B. napus* chromosomes.

This study describes the (i) evaluation of 10 parental lines of different mapping populations, currently being used in the Australian Brassica Germplasm Improvement Program (NBGIP), for Mn^2+^ tolerance, (ii) construction of a high-density linkage map consisting of 7,805 DArTseq markers corresponding to 2,094 unique bin loci in the Darmor-*bzh*/Yudal doubled haploid (DYDH) mapping population, (iii) genetic mapping and identification of molecular markers associated with Mn^2+^ tolerance locus in the DYDH population, (iv) physical localization of the DArTseq sequences linked with Mn^2+^ tolerance locus and (v) identification of putative candidate gene involved in Mn^2+^ tolerance in *B. napus* using *A. thaliana* orthologs/paralogs implicated in Mn^2+^ uptake, accumulation and transport on the reference genomic scaffolds of *B. napus* cv. Darmor-*bzh* (Chalhoub et al., 2014).

## Materials and Methods

### Plant Materials

Ten parental lines of DH populations which are currently being used under the Australian Brassica Germplasm Improvement Program (NBGIP); Skipton, Ag-Spectrum, Tapidor, Ningyou, Darmor-*bzh*, Yudal, Mutu-98-6, RSO94-67, Charlton and Monty (**Table 1**) were evaluated for Mn^2+^ tolerance. Besides, this study involved a DYDH mapping population for genetic tagging of Mn^2+^ tolerance locus. This population was developed previously from an F_1_ cross between the Darmor-*bzh* (winter type; maternal parent of French origin) and Yudal (spring type, paternal parent of Korean origin) at the Institut de Génétique, Environnement et de Protection des Plantes, (INRA), France (Foisset et al., 1995). Seed of DH and parental lines was multiplied in caged tents to ensure purity and avoid any cross-pollination, for different phenotyping and genotyping experiments.

**Table 1:** Mean cotyledon leaf chlorosis, shoot biomass, and leaf area for 10 parental lines of *Brassica napus* mapping populations in nutrient solution culture under Mn stress (+Mn) and control (-Mn) treatments.

Genotype	Country of origin	Leaf chlorosis scale		Fresh shoot biomass (g/plant)		Leaf area (pixel/leaf)	
		+Mn (mean ± SE)	-Mn (mean ± SE)	+Mn (mean ± SE)	-Mn (mean ± SE)	+Mn (mean ± SE)	-Mn (mean ± SE)
Ag-Spectrum	Australia	3.21 ± 0.17^cd^	1.00 ± 0.06^a^	0.74 ± 0.075^bc^	0.97 ± 0.07^de^	88.18 ± 8.51	128.88 ± 8.38
Charlton	Australia	4.00 ± 0.17^e^	1.00 ± 0.06^a^	0.65 ± 0.075^ab^	1.20 ± 0.07^fg^	53.28 ± 8.26	160.91 ± 14.31
Darmor	France	1.63 ± 0.17^b^	1.00 ± 0.06^a^	1.78 ± 0.075^i^	1.59 ± 0.074^hi^	261.21 ± 14.04	198.27 ± 14.04
Monty	Australia	2.74 ± 0.17^c^	1.05 ± 0.06^ab^	0.95 ± 0.075^cde^	1.40 ± 0.07^gh^	90.75 ± 8.38	165.55 ± 8.80
Mutu-98-6	Japan	1.05 ± 0.18^a^	1.00 ± 0.06^a^	1.26 ± 0.075^fg^	1.11 ± 0.07^ef^	137.43 ± 14.04	137.06 ± 14.91
RSO94-67	Australia	4.33 ± 0.17^e^	1.00 ± 0.06^a^	0.45 ± 0.075^a^	0.80 ± 0.07^bcd^	60.70 ± 8.26	108.07 ± 8.51
Skipton	Australia	3.25 ± 0.17^d^	1.18 ± 0.06^b^	0.79 ± 0.705^bcd^	1.19 ± 0.07^fg^	74.70 ± 8.38	145.92 ± 8.38
Tapidor	France/Germany	4.00 ± 0.17^e^	1.00 ± 0.06^a^	0.45 ± 0.075^a^	0.96 ± 0.07^de^	53.28 ± 8.26	181.67 ± 14.31
Ningyou	China	2.96 ± 0.17^cd^	1.00 ± 0.06^a^	0.47 ± 0.075^a^	1.36 ± 0.07^g^	39.17 ± 8.26	192.48 ± 8.26
Yudal	Korea	4.25 ± 0.17^e^	1.00 ± 0.06^a^	0.70 ± 0.075^b^	1.25 ± 0.07^fg^	82.92 ± 8.26	166.86 ± 8.26
	SED	0.24	0.09	9.98E-02			
	LSD (5%)	0.47	0.17	2.08E-01			

*Leaf area was assessed by Image analysis. Alphabetic letters for mean values indicate the significance difference between genotypes.*

### Phenotyping for Mn^2+^ Tolerance

In order to determine the genetic variation for Mn^2+^ tolerance, parental lines of mapping populations were either grown in control (+ Mn treatment, 9 μM MnCl_2_⋅4H_2_O) nutrient solution or in treatment (++ Mn, 134 μM of MnCl_2_⋅4H_2_O) using the nutrient solution culture method as described previously (Raman et al., 2002). A split-plot design of three replicates was followed; with each replicate divided into two main plots (two tubs) to which control (+Mn) or Mn (++ Mn) treatments were randomized. Each tub held a 10 row by 9 range arrangement of cells, with the central range unutilized. Eight seedlings per genotype formed an experimental unit. Treatment experimental units were randomized to blocks of cells arranged as 2 rows by 4 ranges. The experiment was conducted in a temperature controlled plant nutrition laboratory at the New South Wales Department of Primary Industries in Wagga Wagga, Australia (35.0540°S, 147.3485°E), maintained at 20°C ± 2°C.

Two seeds of each *B. napus* genotype were placed for germination in four arrays of 1.5 mL plastic Eppendorf tubes, pre-filled with the ‘Hobby Fill’ fiber (United Bonded Fabrics Pty. Ltd., Melbourne, VIC, Australia). Trays were placed in a tub as shown in Supplementary Figure S1A. Trays were then over-laid on plastic tubs containing 9 L of the nutrient solution which was continuously circulated and aerated as described previously (Raman et al., 2002). The pH of nutrient solution was adjusted to 4.7 daily with either NaOH (100 mM) or HCl (1.0 N), irrespective of treatment imposed. Seeds in trays were covered with a thick black plastic sheet at 20°C for 48 h in continuous darkness. One seedling of similar vigor was subsequently grown per tube in the nutrient solution. Plants were raised under 16 h (day)/8 h (night) photoperiod regime. Experiments were conducted with a light intensity of 122 μmol photons m^-2^s^-1^. After 72 h of Mn treatment, the critical symptoms of Mn^2+^ toxicity as the extent of chlorosis on cotyledon was scored quantitatively as ‘1’ to ‘5’ on daily-basis for 4 days. All scores taken across different days had a very high correlation (*r* = 0.8–0.9), therefore the scores of day 4, were used to characterize different genotypes for tolerance to Mn^2+^ toxicity. A score of ‘1’ indicated a healthy green cotyledon with 0 to ≤10% of cotyledon chlorosis; 2, 3, and 4 scores represented to 10–25%, 25–50%, and 50–75% cotyledon leaf chlorosis, while a score of 5 indicated highly chlorosis covering 75–100% cotyledon area (Supplementary Figures S1B–D). After phenotyping, leaf tissue was collected for genetic analysis.

In order to investigate genetic control for Mn^2+^ tolerance, 196 lines of the DYDH population and its two parental lines were then evaluated for Mn^2+^ tolerance using the basal nutrient solution culture method as described previously (Raman et al., 2002). In addition, two DH lines; ‘MUTU-98-6’ and ‘BLN4101-CO0807-10’; generated by NBGIP at Wagga Wagga, were used as Mn^2+^-tolerant and Mn^2+^-sensitive controls, respectively. This experiment was conducted in a two replicate randomized complete block design in a temperature controlled laboratory in Wagga Wagga. This experiment had a total of 20 plastic tubs (10 tubs per replicate), each tub accommodating 20 genotypes. The trial consisted of 20 row by 20 column tube arrays with each replication consisting of a 20 row by 10 column tube array. Segregation for Mn^2+^ tolerance was scored among DYDH lines as described above.

### Leaf Area and Biomass Measurements

To determine the relationship between Mn^2+^ tolerance/sensitivity and shoot biomass, we cut each seedling from the hypocotyl/root junction after 2 weeks of Mn^2+^ stress and weighed for the fresh shoot biomass from four seedlings of different genotypes (10 parental lines and DYDH lines). We did not measure the root biomass of the seedling as roots were extensively tangled in hydroponic culture and would compromise results (Supplementary Figure S1C). The relationships between Mn^2+^ tolerance and leaf area were further investigated among 10 parental lines under +Mn and ++Mn treatments. Leaf samples were imaged with the standard office printer/scanner (Canon, MP240). Each sample was spread-out and then scanned. RGB images of leaves were converted to 8 bit grayscale ‘jpeg’ file. Bleached leaf samples were saturated in red scale manually. Entire leaf area was measured using the ImageJ program, version 1.42q^2^ and expressed in pixels.

To validate the relationship between Mn^2+^ tolerance/sensitivity and leaf area in the DYDH population, we selected ‘tails’ of DH lines on the basis of the extent of chlorosis. After 14 days of treatment, the first fully expanded leaf from 80 Mn^2+^-tolerant, and 80 Mn^2+^-sensitive seedlings per replication was excised; representing the top 20 Mn^2+^-tolerant (with rating of 1) and the bottom 20 Mn^2+^-sensitive DH lines (with ratings of 4–5). Leaf samples were taken from selected genotypes plus the parental lines of DYDH population and processed within 4 h. Multivariate analysis based on principal components (principal component analysis, PCA) was carried out to understand overall genotypic variation in response to Mn^2+^ stress. Since all phenotypic traits: cotyledon leaf chlorosis (%), shoot biomass (g), and leaf area (pixel) measured under Mn^2+^ stress were on different scales, we standardized the scores; giving equal weight to each variable based on their own mean and variance, and thus correlation matrices were used for PCA.

### DNA Isolation and DArTseq Analysis

Frozen leaf samples taken from the fresh leaves of 2- to 3-week-old seedlings from parental lines and their DH progenies were ground into powder using a Mixer Mill 300 (Retsch, Germany). Genomic DNA was isolated following a standard phenol/chloroform extraction method. Genomic libraries were constructed from different samples using 500 ng DNA/μl for DArTseq marker analysis. DH population along with the parental lines were sequenced with an Illumina 2000 sequencing machine to identify the DArTseq markers. The DArTsoft software version 7.4.7 (DArT P/L) was used to score two types of polymorphisms; (i) DArTseq-SNP and (ii) *silicoDArTs*, also referred to as ‘presence–absence markers’ (2014). DArTseq-SNP markers were scored as ‘1’ for the presence of the reference homozygous SNP allele, ‘0’ for the absence of the reference homozygous SNP allele, “Double null” homozygotes, appearing in the results, due to the absence of a fragment containing the SNP in the genomic representation, were scored as ‘-.’ *silicoDArTs* were scored in a binary fashion, representing genetically ‘dominant’ markers: ‘1’ for the presence and ‘0’ for the absence of a restriction fragment with the marker sequence in the genomic representation. Markers with only ‘1’ or ‘0’ alleles having frequency of zero were discarded. Only high-quality DArTseq markers with a call rate >80% and with reproducibility >90%, were selected for genetic mapping.

### Linkage Map Construction

The genetic linkage map of DYDH population was constructed as described in Raman R. et al. (2016). Linkage map order was checked visually from the graphical representation of recombinant events to ensure the optimal placement of molecular markers. The marker positions were converted to centiMorgans (cM) by applying the Kosambi function (Kosambi, 1944) to the recombination frequencies. The partial linkage map was visualized graphically using the software MapChart (Voorrips, 2002).

### Physical Mapping of DArTseq Markers and Candidate Genes on the *B. napus* Reference Genome

In order to cross-check the genetic positions and chromosomal assignments (A01–A10, C01–C09), marker sequences of the DArTseq were aligned with the published genome assembly of *B. napus* cv. Darmor*-bzh* version 4.1 (Chalhoub et al., 2014) as described previously (Raman H. et al., 2016). Physical positions of DArTseq markers were obtained by BLASTN to search for sequence identities between reference genome sequence and the DArTseq markers. Only the top hits were initially considered to be mapped in the genome using a threshold *E*^-10^ value. BLASTN matches with multiple hits were only considered when that hit was consistent with the ‘assigned linkage group.’ The orthologs/paralogs of candidate genes involved in Mn^2+^ transport and accumulation identified in other plant species were also searched against the ‘Darmor-*bzh*’ reference genome as described above.

### Statistical Analyses

Two linear mixed model approaches, REML (Restricted Estimation Maximum Likelihood using ASreml 3.0) and CLMM (Cumulative Link Mixed Models using ‘ordinal’ in R 3.3.2) were used in the analysis of chlorosis scores of 10 parental lines. ASreml 3.0 (Gilmour et al., 2009) was used to fit a linear mixed model which included fixed effects for treatment, genotype and treatment × genotype and random effects for replicate, mainplot, and experimental unit with residual variance modeled separately for each treatment. Appropriate standard errors (SE) and hence least significant difference (LSD) based on degrees of freedom using the methods of Kenward and Roger (1997) were determined using this model. The most conservative 5% LSD was used for testing between treatments. Results from this analysis were compared with results using ‘CLMM.’

Linear mixed models (ASReml 3.0) were also used to model fresh weight and leaf area with the two models similar to the REML model above but excluding unit effects as there was a single measure per experimental unit but including an autocorrelation structure (AR1 × AR1) to model row and range correlation at the residual level. For fresh weight, a single residual variance was estimated but for leaf area residual variance was found to relate to both treatment and genotype. As an alternative to reporting of leaf area pairwise comparisons based on individual pairwise standard errors of difference (SED), we grouped genotypes into two categories based on the residual variance: group I containing genotypes with residual variance of <2,000 pixel, and group II having residual variance > 2,000 pixel, and then predicted values for leaf area were compared based using 5% LSD within and between groups. For both fresh weight and leaf area, a second model with all terms as random effects was fitted to determine heritability values. The broad-sense heritability (*h*_b_^2^) of Mn^2+^ tolerance was estimated using the equation:

$$\begin{array}{c} h_{b}{}^{2}\;=\;(\sigma_{g}{}^{2}/(\sigma_{g}{}^{2}+\sigma_{e}{}^{2}) \end{array}$$

where σ_g_^2^ is the genetic variance and σ_e_^2^ is the environmental and residual (error) variance. The estimates of genetic and environmental components were obtained from the ASReml 3.0 package.

### Trait-Marker Association Analysis

A dense genetic linkage map of the DYDH population based on 7,805 DArTseq markers corresponding to 2,094 unique loci was utilized for quantitative trait loci (QTL) analysis. Mean cotyledon chlorosis scores were calculated in the ASReml 3.0 package and then used for trait-marker association in the SVS package (Golden Helix, Bozeman, MT, United States). QTL associated with Mn^2+^ tolerance were identified by the logistic marker regression. Markers having LOD score of ≥4 were initially regarded as significant QTL associations. Furthermore, we applied multiple testing corrections: false discovery rate and Bonferroni adjustment [*p* < 2.38777E^-05^ = -log_10_(*p*) of 4.622] for the set of 2,094 markers to establish association between markers and Mn^2+^ tolerance. Manhattan plots revealing trait-marker association on all 19 chromosomes of *B. napus* were generated in the SVS package. The order of DArTseq markers in relation to Mn^2+^ tolerance locus (classified phenotypic data) as tolerant (Darmor-*bzh* type) and sensitive (Yudal type) was rechecked with the RECORD program and presented graphically (Voorrips, 2002; van Os et al., 2006).

## Results

### Canola Varieties Exhibit Significant Genotypic Variation for Tolerance to Mn^2+^

**FIGURE 1:**
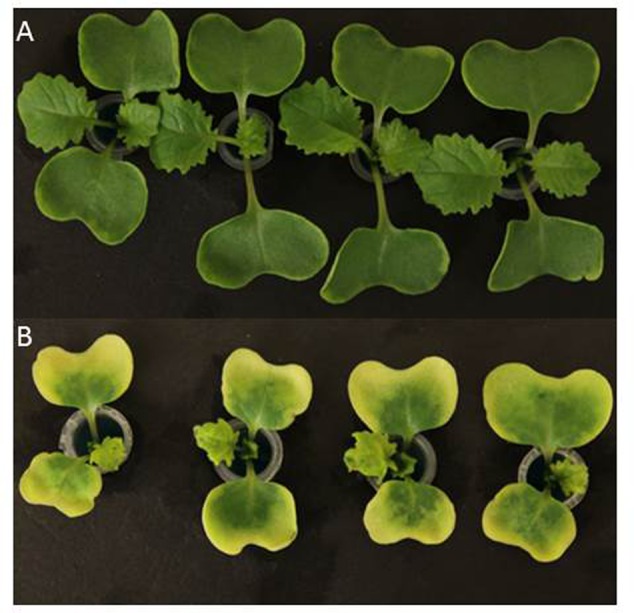
Symptoms of Mn^2+^ toxicity on young canola seedlings (7 days-old) grown in nutrient solution supplemented with MnCl_2_⋅4H_2_O (125 μM). **(A)** Darmor-*bzh*, and **(B)** Yudal.

**FIGURE 2:**
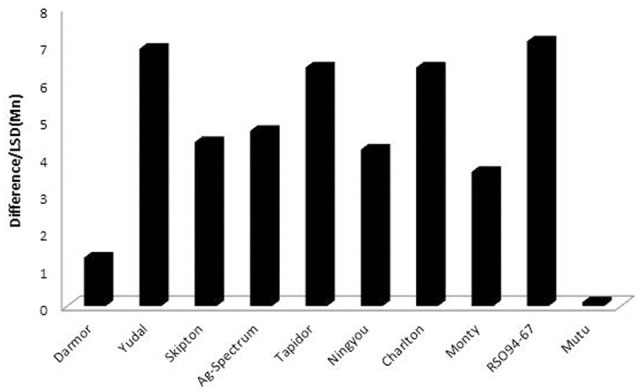
Genetic variation for Mn^2+^ tolerance in *B. napus* genotypes. Tolerance was compared between control (+Mn, 9 μM MnCl_2_⋅4H_2_O) and treatment (++Mn, 125 μM MnCl_2_⋅4H_2_O). The extent of leaf chlorosis was estimated as difference [+Mn mean – control Mn mean/LSD (Mn)].

Ten genotypes exhibited significant differential response to Mn^2+^ stress (125 μM) in the nutrient solution, cotyledon margin chlorosis ranged from 1.05–4.33 scale (**Tables 1, 2**). Among genotypes tested, Mutu-98-6 showed significantly higher tolerance to Mn^2+^ toxicity, as all cotyledon lobes were normal and had very restricted cotyledonary leaf chlorosis (<10% = 1.05 ± 0.18 scale), whereas Yudal, Tapidor, Charlton, and RSO94-67 displayed the least tolerance to Mn^2+^ toxicity (4.33 ± 0.17), characterized by extensive chlorosis/bleaching of up to 90% of the cotyledon (**Figure 1** and Supplementary Figure S1C). Darmor-*bzh* also showed high tolerance to Mn^2+^ (1.63 ± 0.17). Mn^2+^ toxicity symptoms, measured as chlorosis, appeared initially on the cotyledon leaf margins after 5 days of Mn^2+^ stress, followed by extensive chlorosis and cupping of cotyledons. Roots were discolored under ++Mn treatment, however we could not clearly establish whether this is actually due to Mn^2+^ toxicity. Under control treatment, Skipton had significant higher chlorosis than other lines. However, both Skipton and Monty did not differ significantly from each other (**Table 1**). Based on the differential responses of genotypes to Mn^2+^ toxicity, we conclude that Mutu-98-6 and Darmor-*bzh* were unambiguously tolerant as there were no statistical difference [difference/LSD (Mn) = 0.1–1.3] between the control and treatment (**Figure 2**). We rated the other eight varieties; Skipton, Ag-Spectrum, Tapidor, Ningyou, Yudal, RSO94-67, Charlton and Monty, as Mn^2+^-sensitive. To confirm whether genotypic variation revealed by the REML analysis is correct, we analyzed the dataset with the ‘Ordinal’ package using ‘clmm.’ Both results from REML and CLMM were in agreement, suggesting that genetic variation identified by the REML analysis indeed exists for Mn^2+^ tolerance (Supplementary Table S1).

**Table 2:** Restricted maximum likelihood (REML) analysis of per cent leaf chlorosis in 10 parental lines of *B. napus* mapping populations.

Random effect			Fixed effect				
	Variance component	Variance component/SE	Term	*F* statistic	Numerator *df*^A^	Denominator *df*^A^	Probability
Rep	0	0	Mean	3573.15	1	2.5	<0.001
Main plot	0.8888E^-03^	0.42	Treatment	1215.59	1	14.8	<0.001
Unit	0.6976	1.83	Genotype	5.29	9	21.2	<0.001
			Treatment × Genotype	36.58	9	171.9	<0.001

*Residual variance (-Mn): 0.3211E^-*01*^*

*Residual variance (+Mn): 0.613516*

*^A^*df* represents ‘degrees of freedom’*

*Degree of freedom (numerator and denominator) for fixed effects was calculated according to Kenward and Roger (1997). SE, standard error.*

**FIGURE 3:**
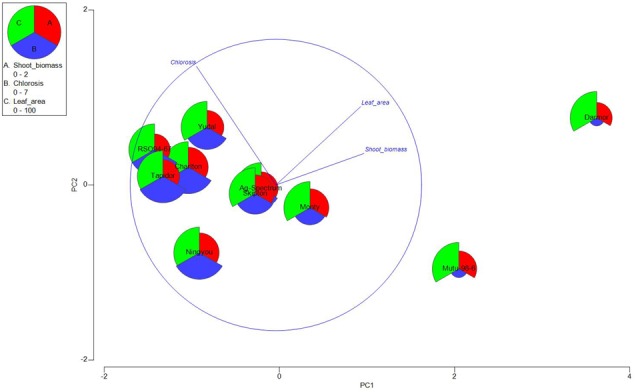
Principal Component Analysis (PCA) of the ten parental lines evaluated for Mn^2+^ tolerance. Genetic variation in Mn^2+^ tolerance was scored on the bases of cotyledon leaf chlorosis, shoot biomass and leaf area. The first two principal components; PC1 and PC2 represent the *X*-and *Y*-axes, accounting for 88.8% and 10.7% of the phenotypic variance, respectively. REML means were normalize using correlation matrices among different component traits: leaf chlorosis, shoot biomass, and leaf area (measured from the second fully expanded leaf).

After scoring for Mn^2+^ tolerance at the cotyledon stage, plants were raised further for 2 weeks. In addition to symptoms on the true leaves, the effect of Mn^2+^ stress on plant (shoot) biomass and leaf area (of first fully expanded leaf) was also measured. REML analysis showed that variation in shoot biomass and leaf area, is genetically determined (Supplementary Table S2). Consistent with previous observations (Wratten and Scott, 1979; Moroni et al., 2003), necrotic spots, interveinal and marginal chlorosis and crinkling of true leaves eventually leading to dark brown necrotic lesions was clearly evident, (Supplementary Figure S2). All Mn^2+^ sensitive genotypes had significantly lower fresh shoot biomass. However, there was no significant decrease in shoot weights and leaf area between ‘control’ and ‘treatment’ in Mn^2+^ tolerant genotypes, Darmor-*bzh* and Mutu-98-6 (**Table 1**), suggesting that Mn^2+^ stress adversely affects plant growth possibly via reducing photosynthetic active leaf area in Mn^2+^ sensitive genotypes. Leaves of several Mn^2+^ sensitive genotypes RSO94-67, Tapidor and Ningyou were highly bleached. Similarly, Mn^2+^ tolerant genotype, Darmor-*bzh* had significantly higher leaf area than other genotypes, irrespective of Mn treatments (5% LSD). Of all genotypes tested, Mutu98-6 had higher leaf area compared to other genotypes and remained unaffected by ++Mn treatment (**Table 1**). The repeatability (*h*_b_^2^) values for leaf chlorosis were higher (90%) compared to shoot biomass and leaf area (65%). Pearson’s correlation coefficient (*r*) was calculated among pairs of test traits, leaf chlorosis was negatively correlated with shoot biomass (*r* = -0.82) and leaf area (*r* = -0.71). Consistent with REML analysis, significant variation in response of genotypes to Mn^2+^ stress was detected with PCA. The normalized REML means for genotypes under Mn^2+^ stress revealed that the first principal component (PC1) account for 88.8 % of the variance (**Table 3**), suggesting that phenotypic variation in response to Mn^2+^ stress was due to major effect of genetic tolerance. The PC2 and PC3 accounted only for 10.5%, and 0.7% of phenotypic variance, respectively. All components; chlorosis, leaf area and shoot biomass contributed equally to PC1 based genotypic variation. Based on these components, all 10 genotypes were assigned to two clusters (I and II). First cluster included Darmor-*bzh* and Mutu genotypes, whereas cluster II consisted rest of the eight genotypes, representing Mn^2+^ tolerant and Mn^2+^ sensitive phenotypes, respectively (**Figure 3**).

**Table 3:** Principal component analysis of normalized means for the extent of leaf chlorosis, shoot biomass and leaf area among 10 parental lines of doubled haploid populations of *B. napus* grown in nutrient solution supplemented with 125 μM of MnCl_2_ 4H_2_O.

	Principal components		
	1	2	3
Eigen values	2.66	0.315	0.022
Per cent variation accounted	88.8	10.5	0.7
**Loading of Mn^2+^ tolerance attributes**			
Leaf chlorosis	–0.545	0.815	–0.199
Shoot biomass	0.604	0.217	–0.767
Leaf area	0.582	0.538	0.610

### A Single Gene Controls Natural Variation for Tolerance to Mn^2+^ Toxicity

**FIGURE 4:**
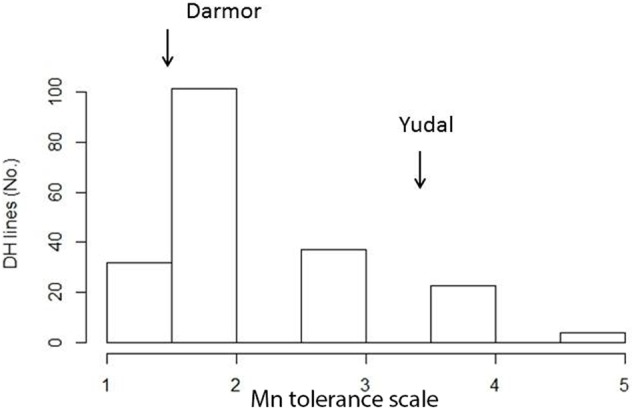
Frequency distribution of Mn^2+^ tolerance scores in the doubled haploid mapping population derived from an F_1_ cross between Darmor-*bzh* and Yudal (DYDH). The phenotypic scores based on the extent of chlorosis of parental lines, Darmor-*bzh* and Yudal are marked with inverted arrows.

The genetic control of Mn^2+^ tolerance was investigated in the DYDH population. Both parental lines of DYDH population showed significant differences for Mn^2+^ tolerance; the extent of leaf chlorosis on Darmor-*bzh* was ∼2.5× lower than that on Mn^2+^-sensitive parent, Yudal (**Table 1**). Phenotypic scores on cotyledon showed the typical skewed normal distribution, suggesting that presumably major qualitative loci controls Mn^2+^ tolerance in this population (**Figure 4**). Some of the DH lines also showed transgressive segregation for Mn^2+^ tolerance. The analysis of variance revealed that the phenotypic variation on Mn^2+^ tolerance is driven mainly due to ‘genotype’ (**Table 4**). The *h*^2^_b_ for Mn^2+^ tolerance in the DYDH population was 65%. These results implied that Mn^2+^ tolerance is a heritable trait in the DY population and, therefore genetic improvement can be made efficiently in the breeding programs.

**Table 4:** ANOVA analysis of Mn^2+^ tolerance, measured as leaf chlorosis in a doubled haploid population from Darmor/Yudal.

	Degree of freedom	DF in the denominator	*F* statistic	Probability
Residual (intercept)	1	1.0	56.370	8.424670e^-02^
DH line	196	87.7	4.893	9.722447e^-15^

### Major Locus for Mn Tolerance, *BnMn^2+^.A09* Is Localized on Chromosome A09 of the *B. napus* cv. Darmor-*bzh*

We constructed a genetic linkage map based on DArTseq markers of the DYDH population (**Table 5**). A total of 7,805 markers, comprising 5,464 *in silico* DArTseq and 2,341 DArTSeq-SNPs, were integrated in the linkage map representing all 19 chromosomes of the A_n_, and C_n_ subgenomes representing a haploid chromosome number of *B. napus* (Supplementary Table S3). Several markers showed distorted segregation ratio, deviating from the monogenic ratio in a DYDH population [overall χ^2^_(1:1)_ = 171.852, *P*_(0.05)_ < 0.0001], consistent with the previous observation (Wang et al., 2011). The length of the linkage groups ranged from 70.94 (A04) to 196.41 (C03) cM. The linkage groups A08, A10, and C07 were shorter compared to others. Of the 19 linkage groups, C03 had the maximum marker density (179), while C09 had the least (64). A_n_ subgenome had the higher number of discrete markers (1,213) compared to the C_n_ subgenome (881). These markers were mapped in 2,094 unique marker bins; covering a total of 2,228.82 cM (Supplementary Table S4) and with an average map density of 0.94 markers per cM (**Table 5**). This genetic linkage map was used further for identification of QTL associated with Mn^2+^ tolerance.

**Table 5:** Summary of markers, and their distribution and density across different linkage groups and subgenomes (A_n_ and C_n_) of the Darmor/Yudal DH population of *B. napus.*

Linkage group	Total mapped markers	*In silico* DArT	DArTseq-SNP	Number of bin marker loci	Genetic map length	Average no. of markers/cM	Average bin markers/cM
A_n_01	373	285	88	115	111.27	3.35	1.03
A_n_02	347	271	76	109	134.08	2.59	0.81
A_n_03	614	404	210	172	147.52	4.16	1.17
A_n_04	349	249	100	93	70.94	4.92	1.31
A_n_05	370	274	96	102	109.08	3.39	0.94
A_n_06	513	338	175	151	125.99	4.07	1.20
A_n_07	520	412	108	120	113.81	4.57	1.05
A_n_08	337	226	111	93	83.57	4.03	1.11
A_n_09	621	425	196	165	130.4	4.76	1.27
A_n_10	517	354	163	93	77.81	6.64	1.20
Subtotal of the A_n_	4,561	3,238	1,323	1,213	1,104.47	4.13	1.10
C_n_01	500	323	177	109	113.97	4.39	0.96
C_n_02	393	295	98	122	150.38	2.61	0.81
C_n_03	594	412	182	179	196.41	3.02	0.91
C_n_04	321	214	107	87	123.39	2.60	0.71
C_n_05	180	123	57	71	121.33	1.48	0.59
C_n_06	238	126	112	77	104.64	2.27	0.74
C_n_07	236	166	70	70	92.7	2.55	0.76
C_n_08	493	351	142	102	107.72	4.58	0.95
C_n_09	289	216	73	64	113.81	2.54	0.56
Subtotal of the C_n_	3,244	2,226	1,018	881	1,124.35	2.89	0.78
**Total of the A_n_C_n_**	**7,805**	**5,464**	**2,341**	**2,094**	**2,228.82**	**3.50**	**0.94**

**FIGURE 5:**
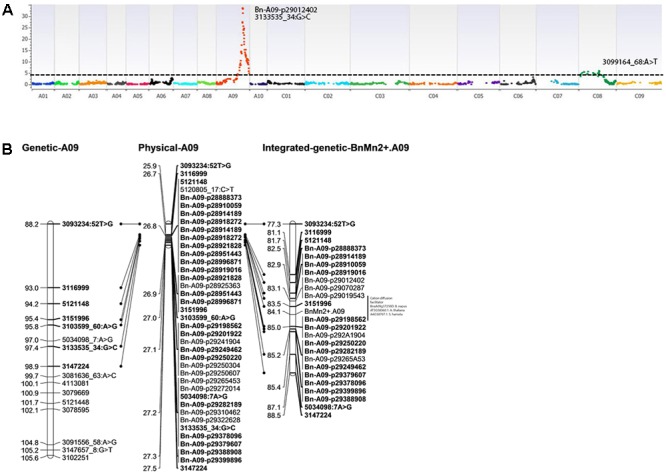
**(A)** Manhattan plots of association between DArTseq markers and Mn^2+^ tolerance scored as per cent leaf chlorosis in the DYDH population. A linkage map based on 2,094 bin-marker loci was used for trait-marker association. The horizontal dotted line represents the Bonferroni-corrected significance threshold of –log_10_(*p*) of 4.6220. Black arrow on the SNP associations indicates the peak signal; **(B)** Map location of a major QTL associated with Mn^2+^ tolerance; *QMn^2+^.wwai-A09* in canola on chromosome A09 in the DYDH population. Localization of DArTseq markers on both genetic and physical map of *B. napus* cv. Darmor-*bzh* (Chalhoub et al., 2014) is shown with dotted lines. Graphical representation was drawn with Record Program. Genetic distances (cM) are shown along the partial linkage map on the *left* side and molecular markers are shown on the *right* side. Vertical lined represents the position of QTL. Candidate genes located between the QTL marker intervals are also shown.

Logistic marker regression analysis revealed 93 markers that showed statistically significant associations (LOD score ≥ 4) with Mn^2+^ tolerance scored on the basis of leaf chlorosis (Supplementary Table S5). Of them, 58 markers were localized on linkage group A09 whereas 24 were mapped on its homologous group C08 (**Figure 5A**). The top two markers, 3151996 (*in silico* DArT) and 3103599_60:A > G (DArTSeq SNP) showed the highly significant association with -log_10_(*p*) score of ≥32.5, suggesting that these markers underlie the genomic region: *BnMn^2+^*.*A09*, for phenotypic variation in Mn^2+^ tolerance on chromosome A09. To validate the association between *BnMn^2+^*.*A09* and DArTseq markers, we targeted 100 kb region of the *BnMn*^2+^.*A09* flanking coordinates 26,812,076–27,047,795 bp, and identified 30 polymorphic Illumina Infinium 60K SNP markers in the DYDH population based of their physical location on the A_n_09 Darmor-*bzh* genome sequence (Clarke et al., 2016). Those polymorphic SNP markers were further tested for their association with phenotypic data (chlorosis scores). Of 30 Illumina SNP markers, Bn-A09-p29012402 located on unassembled portion of the Darmor-*bzh* sequence of chromosome A09-random showed the maximum association (LOD = 34.6) with Mn^2+^ tolerance in the DYDH population (Supplementary Table S6). Eight Illumina SNP markers, which showed the complete linkage with each other (delimited with Bn-A09-p28914189 and Bn-A09-p29087590 markers) also showed highly significant association (LOD = 34.2) with Mn^2+^ tolerance (**Figure 5B**). This marker locus was located within 3.2 kb from the significant DArTseq markers linked with *BnMn^2+^.A09* (Supplementary Table S4). To confirm the trait-marker association results obtained with the SVS package (Supplementary Table S5), we performed interval mapping using R/qtl package (Broman et al., 2003). Two genomic regions: *QMn^2+^.wwai-A09* (LOD = 32.71) on chromosome A09 and *QMn^2+.^wwai-C08* (LOD = 4.01) exhibited significant associations with Mn^2+^ tolerance in a DYDH population. *QMn^2+^.wwai-A09*, flanked by BnA09-p28910059 and 3151996 markers accounted for 41.5% of the variation for Mn^2+^ tolerance, while *QMn^2+^.wwai-C08*, flanked by 5030207_47:T>A and 3111010_34:A>C accounted for 3.9% of the variation. Both QTL were mapped within the same genomic regions that showed significantly associations for Mn tolerance identified with logistic regression approach in the SVS package. At both QTL, the Darmor alleles contributed toward Mn^2+^ tolerance. Both QTL on chromosomes A09 and C08 did not show any significant interaction.

To precisely map this genomic region as a Mendelian locus, we classified DH lines as ‘Darmor-*bzh*’ or ‘Yudal’ type allele based on the phenotypic scores (extent of percent chlorosis): 1–2 as Mn^2+^-tolerant, and 2.1–5 as Mn^2+^-sensitive. Among the DYDH lines, 104 were rated as Mn^2+^-tolerant and 71 were rated as Mn^2+^-sensitive. These segregation ratio showed significant deviation from expected between observed and expected values for a single major locus (χ^2^ = 6.223, *P* = 0.0126). Linkage analysis between phenotype (binary phenotypic data: tolerant/sensitive) and genotypic data (DArTseq and Illumina SNP marker alleles) showed that the two sequence-based markers; 3151996 and BnA09-p29201922 flank the Mn^2+^-tolerance locus in the DYDH population in the order of 3151996 – *BnMn^2+^.A09* – BnA09-p29201922 (**Figure 5B**).

**FIGURE 6:**
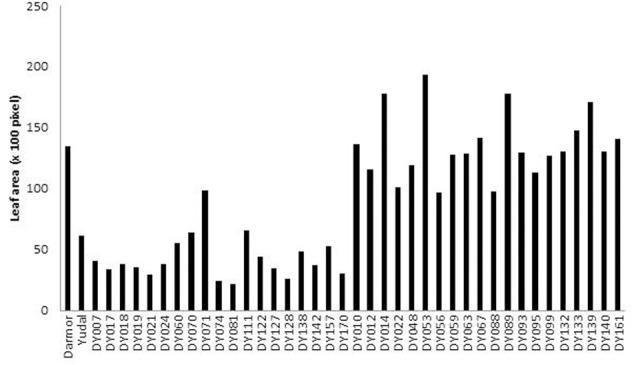
Leaf area of Darmor-*bzh* (tolerant) and Yudal (sensitive) and its 20 extreme Mn^2+^ tolerant and 20 extreme Mn^2+^ sensitive DH progenies of Darmor-*bzh*/Yudal DH population grown in modified nutrient solution supplemented with MnCl_2_⋅4H_2_0 (125 μm). Leaf area was measured with ImageJ software and expressed in pixel.

In order to verify whether the Mn^2+^ stress affect plant growth in the DYDH lines, we measured the shoot biomass and leaf area of 160 individuals of 40 DH lines; representing 20 each extreme tolerant and sensitive DH lines under Mn^2+^ stress. Our results revealed that Mn^2+^ tolerant genotypes had higher fresh shoot biomass compared to sensitive genotype and showed significantly higher correlation (*r* = 0.7). Similarly, a positive correlation (*r* = 0.89) was found between the level of Mn^2+^ tolerance and leaf area among extreme 40 DH lines. Leaf area ranged from 20.4 K pixels (DY081 DH line) to 193.63 K pixels (DY053 DH line); it was higher in tolerant lines than sensitive lines (**Figure 6**). Parental lines; Darmor-*bzh* and Yudal showed the similar trends for both shoot biomass and leaf area.

### Physical Mapping of Significant Marker Loci Linked with *BnMn^2+^.A09* Locus

**FIGURE 7:**
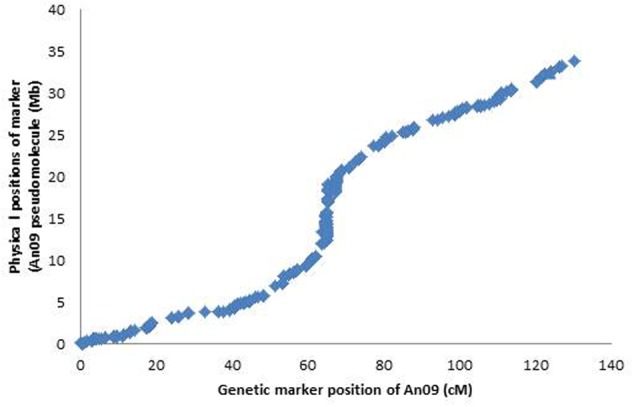
Graphical plot of genetic distance (*Y*-axis, in cM from the linkage map of DYDH population) and physical distance (*X*-axis, in Mb from the genome assembly of Darmor v4.1) for the A09 chromosome of *B. napus* is shown.

We searched the sequence identities including of all 93 markers that showed statistically significant associations with Mn^2+^ tolerance with the reference genome scaffolds of Darmor-*bzh* assembly. Approximately 61% of DArTseq markers (4,741/7,805) could be anchored on the expected pseudomolecules (top hits). Of them (93), 58 markers were localized on the A09 genome scaffold, whereas 24 were mapped on its homologous scaffold; C08 (Supplementary Table S4). Collinearity was observed between the order of markers within the A09 linkage group and the corresponding A_n_09 pseudomolecule of *B. napus* cv. Darmor-*bzh*/*B. rapa* cv. Chiifu, assemblies (**Figure 7**). We could not determine the precise localization of other 11 markers on the Darmor-*bzh* genome assembly (Supplementary Table S4). The highly significant marker; 3151996 (LOD = 32.9) associated with Mn^2+^ tolerance was localized at the physical position, 26,926,623 bp of Darmor-*bzh* sequence within 5 kb of the reference scaffold v4_181 on chromosome An09, and this genomic region is tagged with the scaffold v4_181_291620_BS008304 SNPFSRSO marker (Chalhoub et al., 2014; see Supplementary Table S9).

### Localization of the Putative Candidate Genes Involved in Mn^2+^ Tolerance

To identify candidate genes for Mn^2+^ tolerance between flanking the significant SNP marker intervals flanking the *BnMn^2+^.A09* locus, we searched for sequence identities of genes involved directly or indirectly in Mn^2+^ transport and accumulation in yeast, insects, birds, mammals, and plants, such as natural resistance-associated macrophage protein transporters (*AtNramp1, AtNramp2, AtNramp3*, and *AtNramp4);* divalent metal transporters; phosphate transporter 1; ZIP (Zn-regulator transporter/iron-regulated transporter (ZRT/IRT1); endoplasmic reticulum Ca^2+^- and Mn^2+^ transporting P-type ATPase; tonoplast-localized cation/H^+^ antiporter; the cation exchanger, *AtCAX2*; metal transporter protein 1; vacuolar-localized cation diffusion facilitator/cation efflux family (*ATMTP11, shMTP1-4, ATMTP8, shMTP8*); yellow stripe-like (*AtYSL1*) transporter; and oligopeptide transporter-like protein (*AtOPT3*), reviewed by Delhaize et al. (2003; Pittman, 2005; Mills et al., 2008), against the reference Darmor-*bzh* scaffolds. The physical positions of these genes were filtered based on their physical positions on the Darmor-*bzh* genome sequence (Supplementary Table S7). Of the 21 genes, a putative candidate gene, *BnaA09g37250D* annotated for cation efflux family protein, was identified in the *BnMn^2+.^A09 region* (at nucleotides 26,817,777 to 26,820,251 bp). This region was delimited by a DArTseq SNP marker, 5120805_17:C>T that showed the highly significant association with *BnMn^2+^.A09* locus. *BnaA09g37250D* gene (in ‘Darmor-*bzh’* assembly) also showed significant sequence similarities with that of *A. thaliana* (AT3G58060.1) and *B. napus* cv. ZY11 (LOC106368173, GWSAtT00020294001) that encode for cation efflux family and putative metal tolerance proteins, respectively (**Table 6**). *BnMn^2+^.A09* locus was mapped in the vicinity of an ortholog of a cation diffusion facilitator gene identified in *Arabidopsis thaliana* (*MTP8*) and in a tropical forage legume, *Stylosanthes hamata* [*shMTP8*, AAO38707.1] which were mapped ∼ 933 bp apart. Annotation of *B. napus* reference genomes (Darmor-*bzh* and ZY11) suggested that cation efflux transporter is a likely candidate gene for Mn^2+^ tolerance in the DYDH population. Other genes were either mapped far away from the Mn^2+^ tolerance locus on A09 or mapped on other chromosomes of *B. napus* genome, where major genetic effects were not detected (Supplementary Table S7). For example, four orthologs of *Arabidopsis* gene; *AtMTP11* (AT2G39450.1) encoding a Golgi-localized manganese transporter (Delhaize et al., 2003; Peiter et al., 2007)) were localized on chromosomes A04/C04, A05_random, and C08, but not in the vicinity of *BnMn^2+^.A09*. One of the *ATOPT3* orthologs was located about 6.4 Mb apart from the highly significant SNPs; 3093077_20:T>G, and 3183817 at the *BnMn^2+^*.*C08* locus (Supplementary Table S6). Homoeolog of BnaA09g37250D *B. napus* gene encoding for cation efflux was also located 8.5 Mb from the significant associated markers on chromosome C08. The orthologs of *AtNRAMP1* gene involved in ion transport (Cd, Fe, and Mn^2+^) and homeostasis, were localized on group A02/C02, and A07/C06 chromosomes (BLAT score 1,075–1,272) but showed low identity scores with the genomic region on chromosome A09. This region was located approximately 13 Mbp apart from *BnMn^2+^*.*A09* locus. No sequence similarity was found against divalent metal ion transporter gene, SMF1 of *Saccharomyces cerevisiae* (NM_001183376.1).

**Table 6:** Candidate genes ascribed to map in the vicinity of markers linked with *BnMn^2+^*.*A09* locus for tolerance to Mn^2+^ toxicity in the Darmor-*bzh*/Yudal DH population.

Marker	Physical map position of marker on the Darmor reference genome	Candidate gene	Species	Physical map position of candidate gene on the Darmor reference genome (bp)	Distance (bp) between physical positions of marker and candidate gene	Annotation of gene
Bn-A09-p28888373	26,843,581	LOC106368173	*B. napus* cv. ZY11	26,817,777 – 26,820,251	3,991	Putative metal tolerance protein C3
5120805_17:C>T	26,813,149	LOC106368173	*B. napus* cv. ZY11	26,817,777 – 26,820,251	4,628–7,102	Putative metal tolerance protein C3
		BnaA09g37250D	*B. napus* cv. *Darmor-bzh*	26,817,835 – 26,819,800	4,686–6,651	Cation efflux family protein (GWSAtT00020294001)
		AT3G58060.1	*A. thaliana*	26,817,908 – 26,819,774	4,759–6,625	Cation efflux family protein
		AAO38707.1	*Stylosanthes hamata*	26,818,477 – 26,819,469	5,328–6,320	Cation diffusion facilitator 8

*For simplicity, we provided the smallest physical distance from significant SNP association. Details are given in a Supplementary Table S7.*

## Discussion

In this study, we aimed to understand the extent of genetic variation among the parental lines of *B. napus* NBGIP populations, genetic control of Mn^2+^ tolerance and identification of gene-specific molecular markers associated with a genomic region underlying phenotypic variation for Mn^2+^ tolerance. Phenotypic assay used herein enabled us to characterize different genotypes of *B. napus*, and determine the genetic control for tolerance to Mn^2+^ toxicity. Lower concentration of Mn^2+^ (0.01 to ∼400 μM) is required for various biochemical processes such as oxygen evolution in photosynthesis, detoxification of oxygen-free radicals (Fox and Guerinot, 1998), and lignin and suberin biosynthesis (Marschner, 1995). However, higher levels of Mn^2+^ cause toxicity to plants growing on acidic soils. In nature, some plant species have evolved different mechanism to cope Mn stress, via exclusion of Mn from root/shoots, conversion of metal to metabolically inactive compound (e.g., Mn^2+^-chelate complex) or sequestration of the Mn^2+^ ions in various organelles (Horiguchi, 1988). Our results showed that even 9 μM of MnCl_2_ (present in control solution), is detrimental to plant growth of ‘Skipton’ (**Table 1**). While, the French cultivar; Darmor-*bzh* could tolerate higher levels of MnCl_2_ (125 μM). Our preliminary data based on unreplicated trials suggest that Mn^2+^ tolerance also exists in the French cultivar, Jet Neuf (original data not shown), which is one of the parents of Darmor-*bzh* (Pilet et al., 2001). This suggests that the genotypic variation for tolerance to Mn^2+^ toxicity exists in European germplasm. Previously, Mn^2+^ tolerance was reported in *B. napus* varieties originated from Japan (‘Mutu’), Canada (‘91-215-3’ and ‘Tower’) and Australia (‘Wesreo’) (Moroni et al., 2003). Differential genotypic response to Mn^2+^ stress suggest that ‘Mutu’ is more tolerant compared to ‘Darmor-*bzh*’, and allelic diversity is likely present at Mn^2+^ tolerance locus. Further research is required to establish whether multiple genes control Mn^2+^ tolerance in *B. napus*, as reported in other crops, e.g., wheat (Raman et al., 2005; Ryan et al., 2009) or superior alleles are only present at *BnMn^2+^*.*A09* locus. No heterogeneity for Mn^2+^ tolerance was observed among DH lines utilized in this study and this has made genetic analysis easier. In previous studies, several accessions were sourced from ATFCC (now Australian Grains Genebank), which showed heterogeneous response to Mn stress (Moroni et al., 2003). We further verified that Mn^2+^ toxicity affects plant growth possibly via affecting photosynthetic machinery as evident by extensive leaf chlorosis and reduction in leaf area.

Genetic analysis of the DYDH population revealed that a major locus controls Mn^2+^ tolerance, consistent with a previous study conducted in an intercross population from Mutu98-6/RSO94-67 (McVittie et al., 2011). For the first time, we further show that two loci for Mn^2+^ tolerance could be localized in homoeologous A09/C08 regions: one on C09 with a major effect and one on C08 with a minor effect. Further research is required to establish the role of C08 homolog in the natural variation for Mn^2+^ tolerance in diverse *B. napus* germplasm. We observed a high level of segregation distortion especially in the DYDH population. Such segregation distortions were reported for Al^3+^ in many *B. napus* mapping populations (Wang et al., 2011; Raman et al., 2013) and may be related to differential response to tissue culture.

The synteny between Darmor-*bzh* scaffolds and *A. thaliana* genes^3^ has facilitated the identification of *B. napus* orthologs in the present study. We found that BnaA09g37250D *B. napus* gene; ortholog to *MTP8* in *A. thaliana* and to *shMTP8* in *S. hamata*, both implicated in the membrane trafficking of Mn^2+^ into the vacuole, map in the vicinity of *BnMn^2+^*.*A09* locus. This suggests that cation efflux (diffusion) facilitator gene is a likely candidate which may control genetic variation for Mn^2+^ tolerance in the DYDH population. Alignment of amino acid sequences of *MTP8* gene from *S. hamata* and other species showed higher sequence identities with *Glycine max* and *A. thaliana* compared to *B. napus* (Supplementary Figure S3). This suggests that *MTP8* gene is diverse, but conserved in different plants. Other broad spectrum Mn^2+^–Ca^2+^ and Mn^2+^–Fe^2+^ transporters; *AtNRAMP1* and *AtMTP11* implicated in Mn^2+^ tolerance via sequestering Mn into internal organelles (Pittman, 2005), do not map in the vicinity of the *BnMn^2+^*.*A09* locus. Therefore, it is unlikely that those genes are involved in Mn^2+^ tolerance in the DYDH population.

The DYDH population also show segregation for a range of agronomic traits such as flowering time, plant height, seed quality traits, and qualitative and quantitative resistance to *L. maculans* causing blackleg disease (Pilet et al., 2001; Delourme et al., 2004, 2006; Huang et al., 2009). Tagging of the *BnMn^2+^.A09* gene associated with Mn^2+^ tolerance, performed in this study, would allow developing superior canola varieties faster for selection for Mn^2+^ tolerance, in addition to other favorable Darmor-*bzh* alleles described above. Molecular markers could also be used to characterize allele(s) for Mn^2+^ tolerance in *B. napus* as well as related species such as *Brassica rapa* and *Brassica juncea*. Mn^2+^ toxicity symptom characterized by leaf crinkling and chlorosis may be confused with Ca and Fe deficiencies, respectively and molecular markers identified herein, may provide a direct method to screen germplasm for Mn^2+^ tolerance. Furthermore, high *h*_b_^2^ values (65–95%) coupled with a simple inheritance of Mn^2+^ tolerance mediated by a single locus, makes this trait amenable for selection and breeding.

Several *B. napus* QTL for agronomic importance such as seed size, seed number per pod, seed weight, pod number, pod length, grain yield, acid detergent fiber and lignin content, erucic acid content, flowering time, plant height, and pod shatter resistance have been mapped on chromosome A09 in *B. napus* diverse populations (Liu et al., 2013, 2016; Li F. et al., 2014; Li N. et al., 2014; Raman et al., 2014; Schiessl et al., 2015; Shi et al., 2015; Geng et al., 2016; Körber et al., 2016; Raman R. et al., 2016). Some of these genomic regions, for example seed weight QTL are located close (1 Mb) to the *BnMn^2+^.A09* locus. Therefore, it is important to consider the positions of those QTL, so that strategies for marker-assisted selections could be developed in pragmatic breeding programs to raise production on acid soils.

## Conclusion

Here, we show that the tolerance to Mn^2+^ is mainly mediated by a major locus, *BnMn^2+^*.*A09* that maps in the vicinity of a Cation Diffusion Facilitator gene on chromosome A09 in the Darmor-*bzh*/Yudal population. Newly identified genetic resource (Darmor-*bzh*) of tolerance to Mn^2+^ toxicity, combined with tightly linked SNP markers to Mn^2+^ tolerance locus *BnMn^2+^.A09*, would provide an invaluable genetic tool in developing Mn^2+^ tolerant canola varieties for adaptation to acid soils. We anticipate that genetic variation for Mn^2+^ tolerance could be harnessed using naturally occurring diversity present in *B. napus* and molecular markers. Breeding for Mn^2+^ tolerance may enhance grain yield for achieving greater return on investment to growers and possibly may facilitate the expansion of canola cultivation on acidic soils. Validation of SNP markers for their usefulness to predict Mn^2+^ tolerance in a diverse *B. napus* germplasm is in progress.

## Author Contributions

HR coordinated, supervised and designed this study. BM and HR phenotyped parental lines of NBGIP mapping populations, and of DYDH lines. HR, RR, and BO analyzed the data, BO and RR designed experimental designs for phenotyping parental and DYDH lines, RD provided the DYDH population and Illumina SNP data, HR wrote the manuscript and all the authors have commented, edited and approved the final manuscript.

## Conflict of Interest Statement

The authors declare that the research was conducted in the absence of any commercial or financial relationships that could be construed as a potential conflict of interest.

## Footnotes

1 http://faostat3.fao.org/home/index.html

2 https://imagej.net/

3 http://www.genoscope.cns.fr/brassicanapus/
