# Supplementary material for: A Major Locus for Manganese Tolerance Maps on Chromosome A09 in a Doubled Haploid Population of Brassica napus L
Source: Front Plant Sci. 2017 Dec 12;8:1952. doi: 10.3389/fpls.2017.01952 (PMC5733045; doi:10.3389/fpls.2017.01952)
Supplement: Supplementary file 2 [file Table_2.docx]

Supplementary Table 2

Supplementary Table 2a: Restricted maximum likelihood (REML) analysis of shoot biomass of ten parental lines of *B. napus* mapping populations, grown in control (-Mn) and + Mn (125µM) nutrient solution. Degree of freedom (numerator and denominator) for fixed effects was calculated according to Kenward and Roger (1997).

| Random  effect | | | Fixed effect | | | | |
| --- | --- | --- | --- | --- | --- | --- | --- |
|  | Variance component | Variance/  Standard error | Term | F statistic | Numerator | Denominator | Probability |
| Rep | 0 | 0 | Mean | 1864.70 | 1 | 4.0 | < 0.001 |
| Main plot | 0 | 0 | Treatment | 75.45 | 1 | 9.9 | < 0.001 |
| Spatial AR1 ranges x AR1 rows | 0.16 | 0.21 | Genotype | 36.51 | 9 | 22.2 | < 0.001 |
|  |  |  | Treatment x Genotype | 11.41 | 9 | 26.1 | < 0.001 |
|  |  |  |  |  |  |  |  |
| Residual variance | 0.176E^-01^ | 4.18 |  |  |  |  |  |

Supplementary Table 2b: Restricted maximum likelihood (REML) analysis of leaf area of ten parental lines of *B. napus* mapping populations, grown in control (-Mn) and + Mn (125 µM) nutrient solution. Degree of freedom (numerator and denominator) for fixed effects was calculated according to Kenward and Roger (1997).

| Random  effect | | | Fixed effect | | | | |
| --- | --- | --- | --- | --- | --- | --- | --- |
|  | Variance component | Variance/  Standard error | Term | F statistic | Numerator | Denominator | Probability |
| Rep | 10.38 | 0.45 | Mean | 956.65 | 1 | 2.0 | < 0.001 |
| Main plot | 0.160E-05 | 0 | Treatment | 316.82 | 1 | 31.1 | < 0.001 |
| unit | 57.52 | 1.01 | Genotype |  |  |  | < 0.001 |
|  |  |  | Treatment x Genotype | 41.5 | 9 | 14.98 | < 0.001 |
| Group 1 (<2000 pixels) | 1093.15 |  |  |  |  |  |  |
| Group 2(>2000 pixels) | 4190.54 |  |  |  |  |  |  |
