# Supplementary material for: A Major Locus for Manganese Tolerance Maps on Chromosome A09 in a Doubled Haploid Population of Brassica napus L
Source: Front Plant Sci. 2017 Dec 12;8:1952. doi: 10.3389/fpls.2017.01952 (PMC5733045; doi:10.3389/fpls.2017.01952)
Supplement: Supplementary file 8 [file Presentation_1.pptx]

## Slide 1
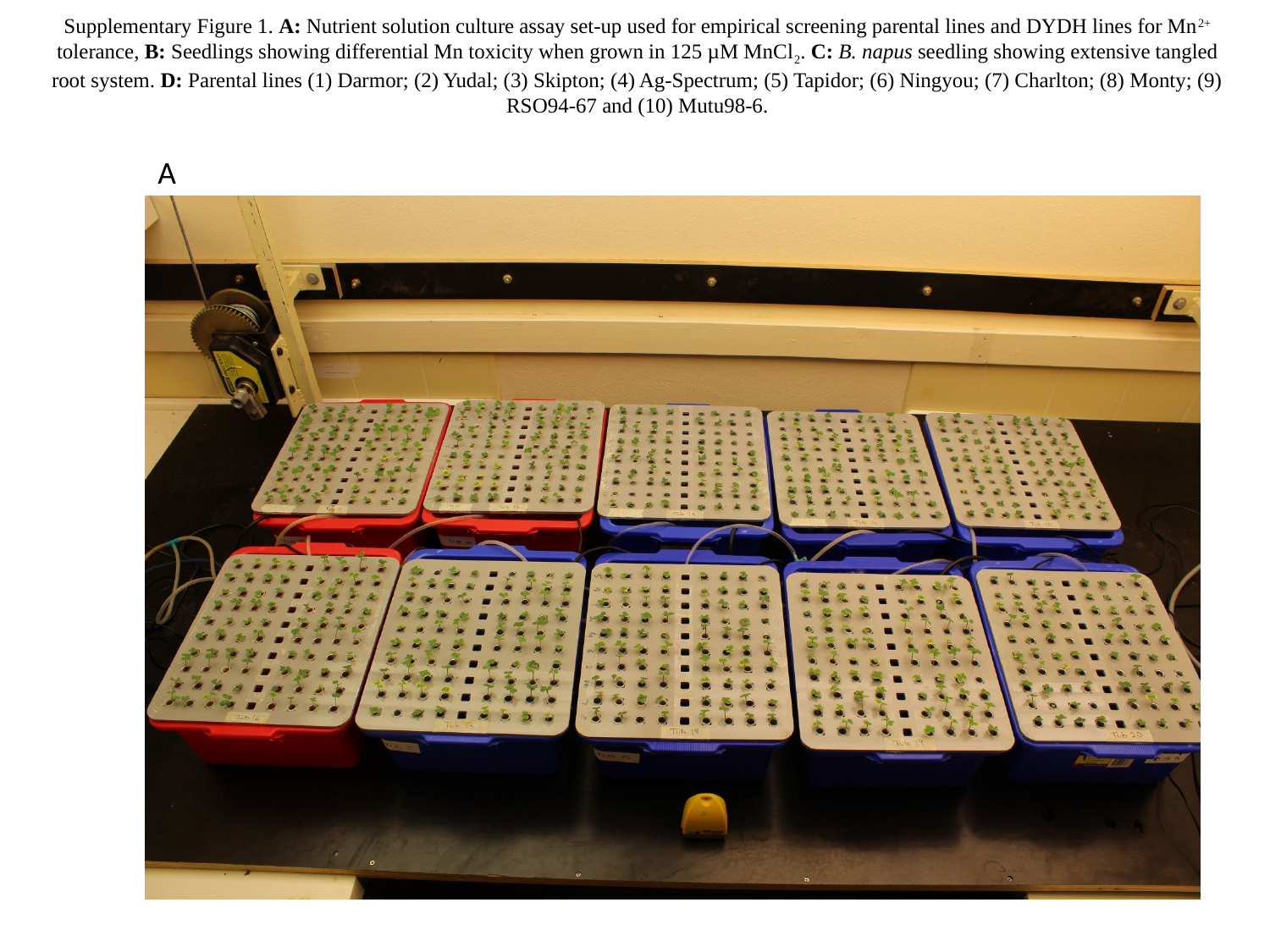

Supplementary Figure 1. A: Nutrient solution culture assay set-up used for empirical screening parental lines and DYDH lines for Mn2+ tolerance, B: Seedlings showing differential Mn toxicity when grown in 125 µM MnCl2. C: B. napus seedling showing extensive tangled root system. D: Parental lines (1) Darmor; (2) Yudal; (3) Skipton; (4) Ag-Spectrum; (5) Tapidor; (6) Ningyou; (7) Charlton; (8) Monty; (9) RSO94-67 and (10) Mutu98-6.
A

## Slide 2
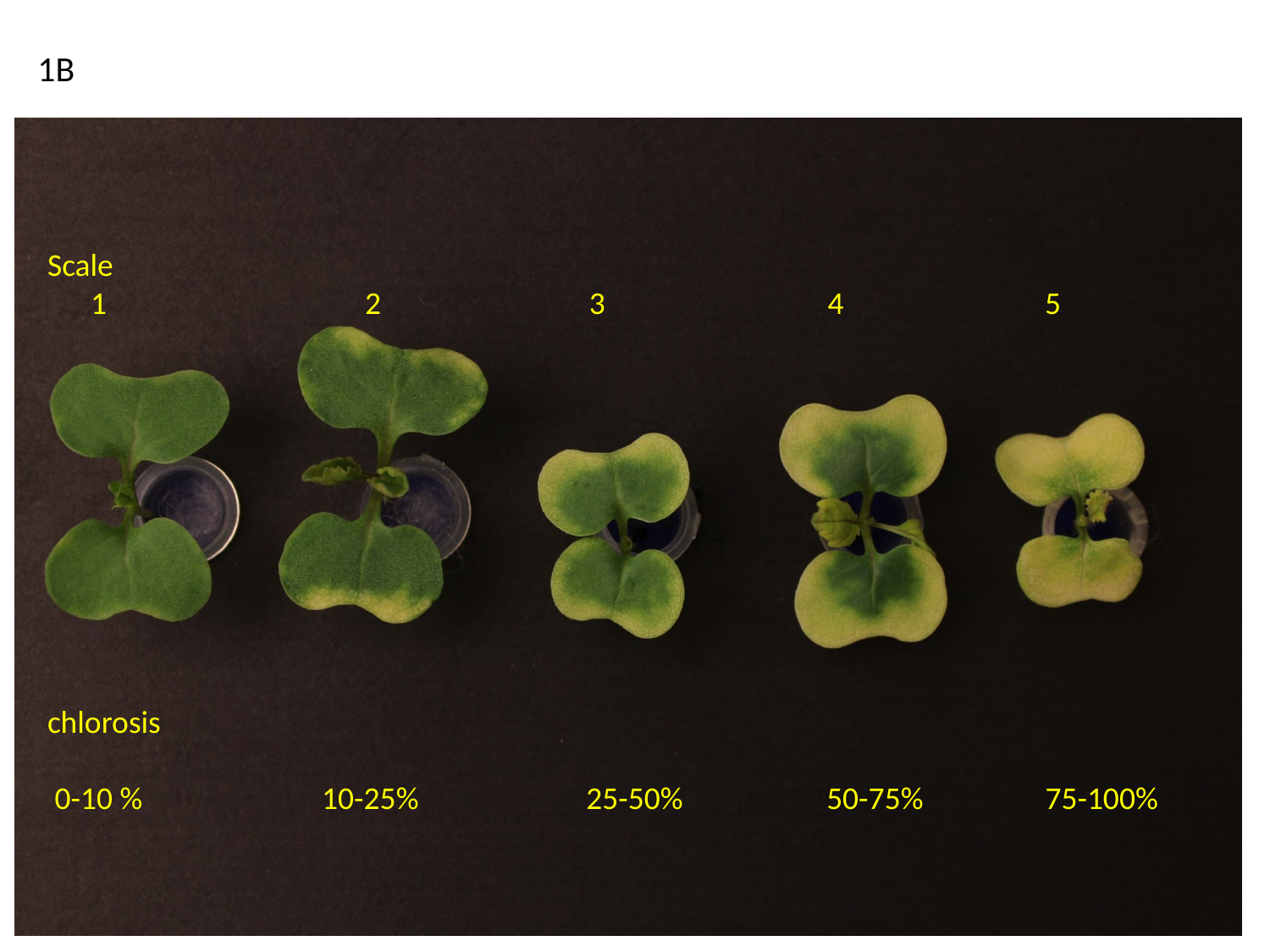

# 1B
Scale
 1 2 3 4 5
chlorosis
 0-10 % 10-25%	 25-50% 50-75% 75-100%

## Slide 3
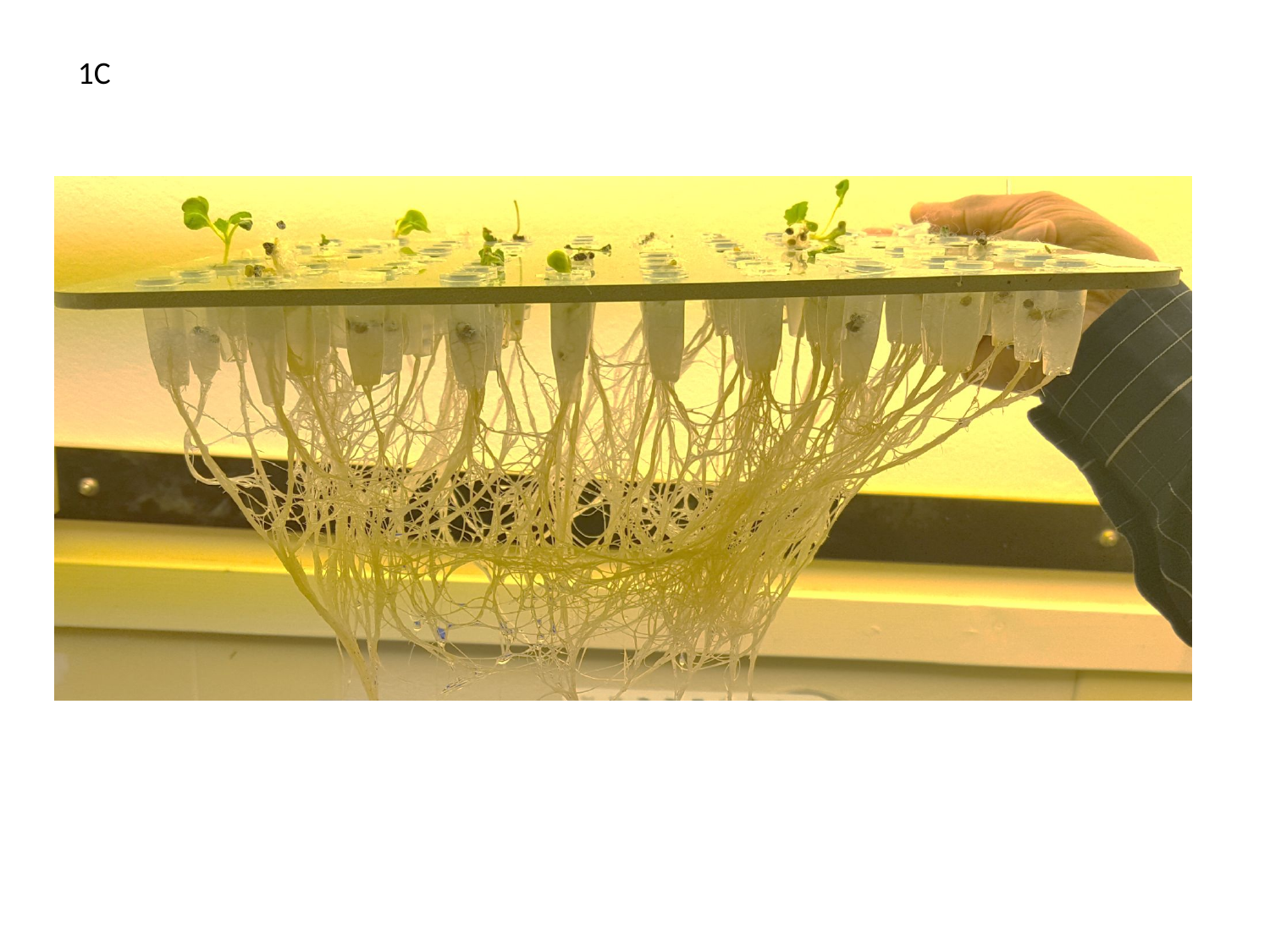

1C

## Slide 4
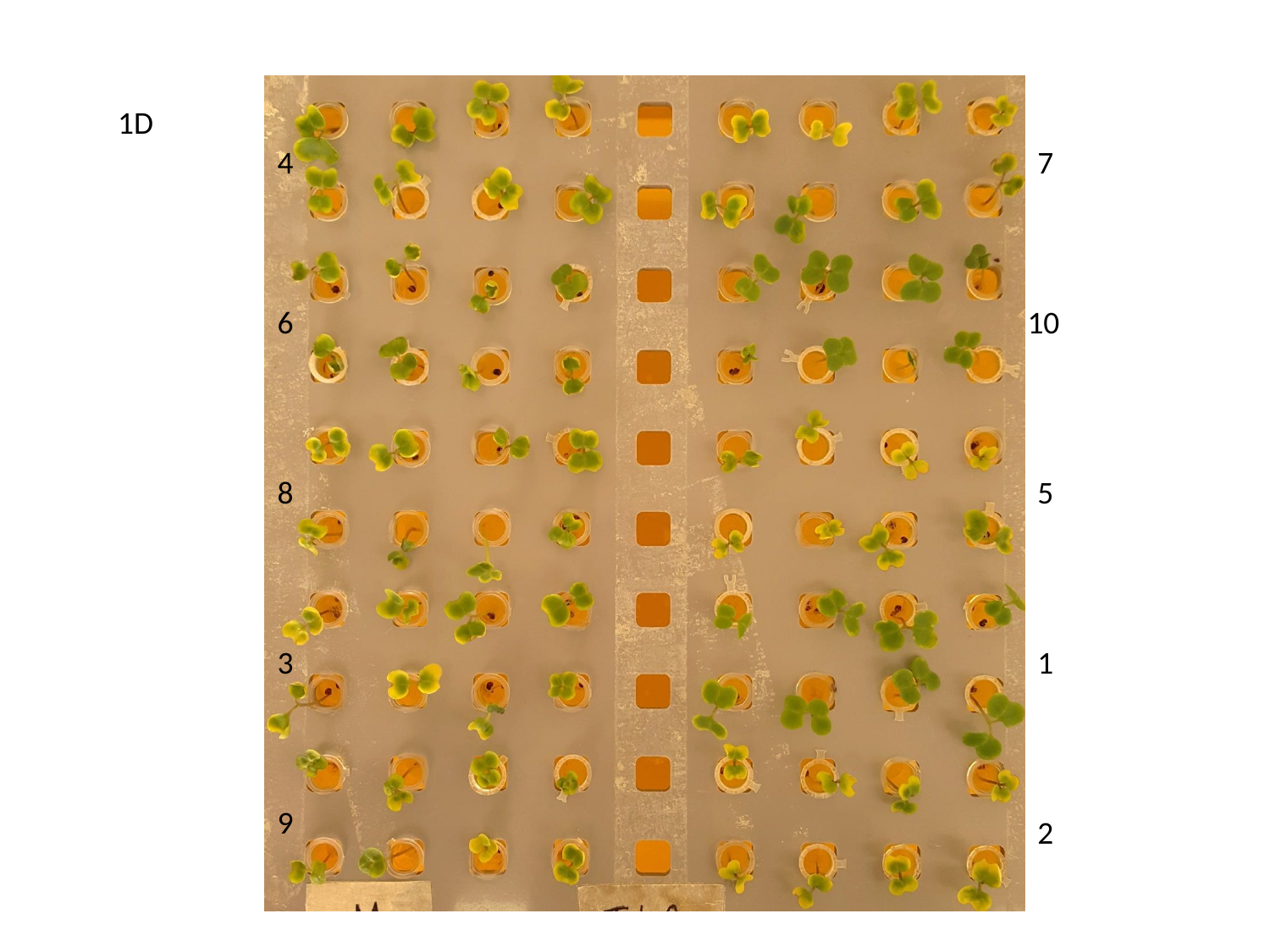

4
7
6
10
8
5
3
1
9
2
1D
