# Supplementary material for: A Major Locus for Manganese Tolerance Maps on Chromosome A09 in a Doubled Haploid Population of Brassica napus L
Source: Front Plant Sci. 2017 Dec 12;8:1952. doi: 10.3389/fpls.2017.01952 (PMC5733045; doi:10.3389/fpls.2017.01952)
Supplement: Supplementary file 9 [file Presentation_2.pptx]

## Slide 1
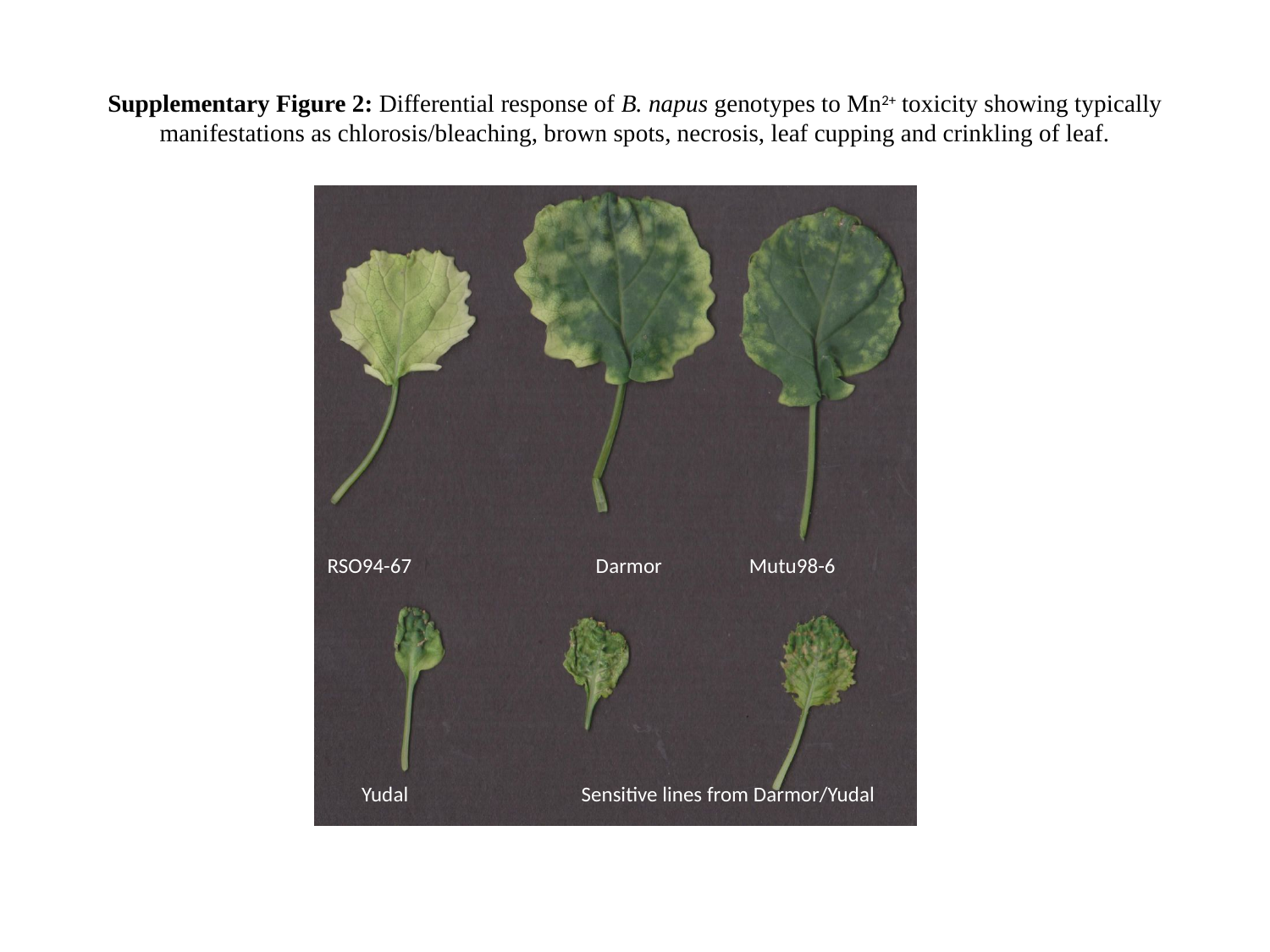

# Supplementary Figure 2: Differential response of B. napus genotypes to Mn2+ toxicity showing typically manifestations as chlorosis/bleaching, brown spots, necrosis, leaf cupping and crinkling of leaf.
RSO94-67 	 Darmor Mutu98-6
 Yudal		Sensitive lines from Darmor/Yudal
