# Supplementary material for: A Major Locus for Manganese Tolerance Maps on Chromosome A09 in a Doubled Haploid Population of Brassica napus L
Source: Front Plant Sci. 2017 Dec 12;8:1952. doi: 10.3389/fpls.2017.01952 (PMC5733045; doi:10.3389/fpls.2017.01952)
Supplement: Supplementary file 10 [file Presentation_3.pptx]

## Slide 1
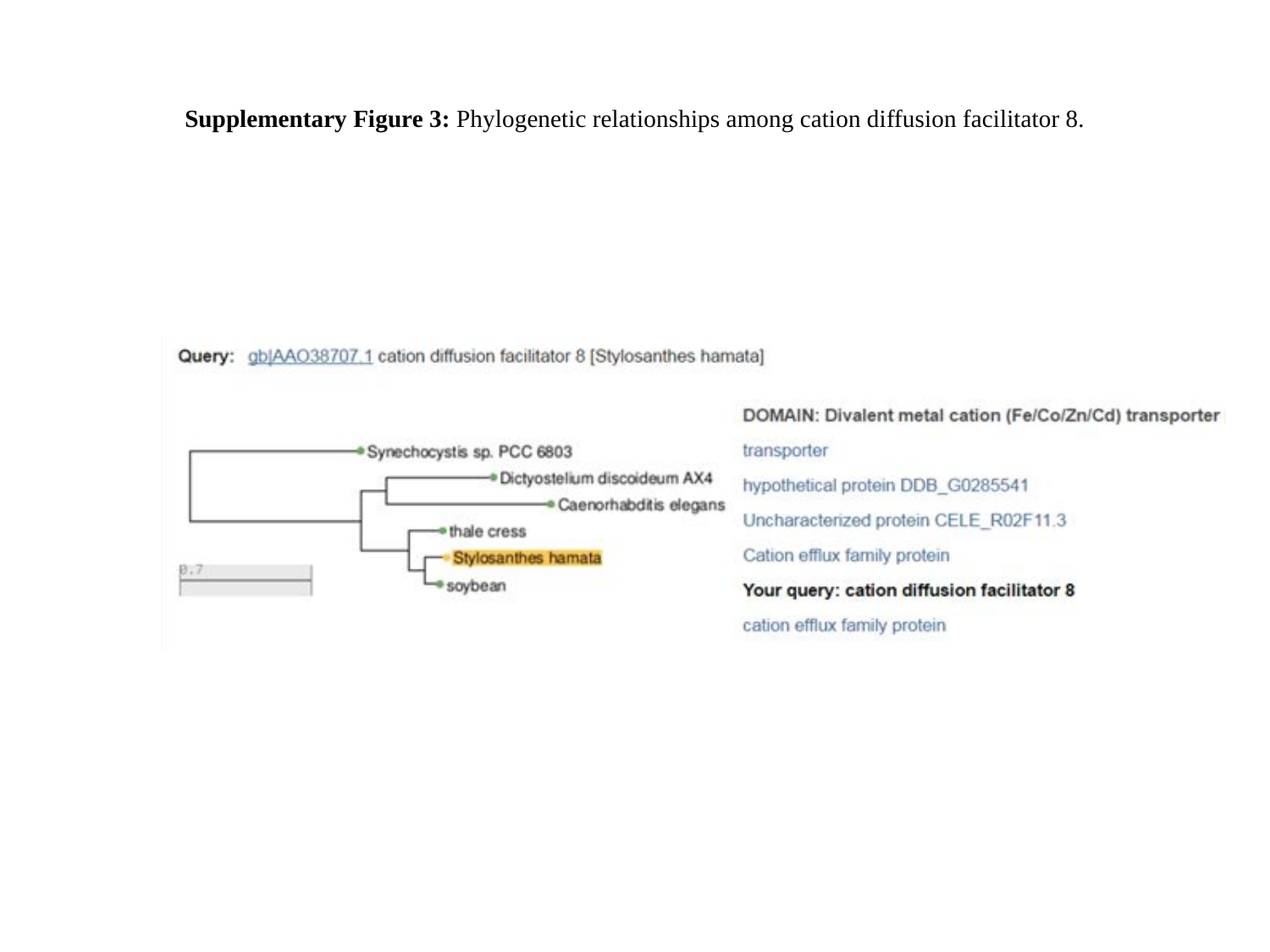

# Supplementary Figure 3: Phylogenetic relationships among cation diffusion facilitator 8.
